# The Dynamics of Developmental and Tumor Angiogenesis—A Comparison

**DOI:** 10.3390/cancers4020400

**Published:** 2012-04-11

**Authors:** Yi Jin, Lars Jakobsson

**Affiliations:** Department of Medical Biochemistry and Biophysics, Karolinska Institutet, Stockholm SE-17177, Sweden; E-Mail: Yi.jin@ki.se

**Keywords:** angiogenesis, VEGF, sprouting, tip cell, vascular dynamics, tumor, cancer, blood vessels, remodeling, tumor stroma

## Abstract

The blood vasculature in cancers has been the subject of intense interest during the past four decades. Since the original ideas of targeting angiogenesis to treat cancer were proposed in the 1970s, it has become evident that more knowledge about the role of vessels in tumor biology is needed to fully take advantage of such strategies. The vasculature serves the surrounding tissue in a multitude of ways that all must be taken into consideration in therapeutic manipulation. Aspects of delivery of conventional cytostatic drugs, induction of hypoxia affecting treatment by radiotherapy, changes in tumor cell metabolism, vascular leak and trafficking of leukocytes are affected by interventions on vascular function. Many tumors constitute a highly interchangeable milieu undergoing proliferation, apoptosis, and necrosis with abundance of growth factors, enzymes and metabolites. These aspects are reflected by the abnormal tortuous, leaky vascular bed with detached mural cells (pericytes). The vascular bed of tumors is known to be unstable and undergoing remodeling, but it is not until recently that this has been dynamically demonstrated at high resolution, facilitated by technical advances in intravital microscopy. In this review we discuss developmental genetic loss-of-function experiments in the light of tumor angiogenesis. We find this a valid comparison since many studies phenocopy the vasculature in development and tumors.

## 1. Introduction

### 1.1. The Tumor Vasculature

Tumor growth requires a supply of oxygen and nutrients, normally supported via blood vessels. Tumor cells positioned near a capillary are supported until the increasing volume limits sufficient diffusion. Cells that for genetic reasons can sustain cellular functions at low oxygen levels might be selected but in order to proliferate further, the supply of nutrients, hence the close vicinity to capillaries has to be maintained. When the oxygen level drops vascular endothelial growth factor-A (VEGFA, also denoted vascular permeability factor) will be secreted from the tumor cells (reviewed in [1]). This growth factor will induce endothelial cell (EC) proliferation, migration and vascular leakage that give rise to an expansion of the vascular bed. This expansion may occur through several conceptually different processes: sprouting angiogenesis (migration and proliferation of EC), intussusception (splitting of vessels by pillar formation), co-option (tumor cells adopt the preexisting vasculature), vascular mimicry (tumor cells and other stromal cells form channels) and possibly looping (new vascular loops are forming, driven by tension forces) and *in situ* differentiation of cancer stem-like cells [2,3,4,5,6]. Because of the technical challenges of studying these dynamic processes, we know little about their respective contribution to vascular growth in tumors [7]. Regardless of how vessels are formed, it is commonly accepted that the vasculature of tumors is different from that of the surrounding tissue. In comparison with normal well organized functional vessels they are often tortuous, pericyte-deficient and leaky, with irregular diameter and altered arterial to venous hierarchy [8]. Some of these properties (discussed below) are commonly seen in developmental angiogenesis—A process that has been intensely studied, providing deep knowledge on cellular behavior, especially in sprouting [9,10]. Because of these shared properties between the angiogenic vasculature in development and the tumor vasculature it might be informative to apply the knowledge gained from developmental studies on the process of tumor angiogenesis. Below we focus on molecules that have central roles during initiation, migration and elongation of new branches in the process of sprouting angiogenesis. It is important to point out that their requirement for guided sprouting does not exclude a role during the alternative modes of vascular expansion. Within this review we use detailed information from developmental gene loss-of-function (LOF) studies to further understand the process of tumor angiogenesis. We find this a valid comparison since many LOF studies phenocopy the vasculature in development and tumors (Table 1, and discussed below).

**Table 1:** Comparison of vascular phenotypes in development and cancer following gene modifications or drug interventions.

	Genetic Deletion		Drug/Ab Intervention	
Gene	Developmental phenotype	Tumor phenotype	Developmental phenotype	Tumor phenotype
*Vegfa*	*Vegfa^−/−^*: lethal at E9.5 due to abnormal vascular development. *Vegfa^+/−^*: lethal at E11.5 due to abnormal vascular development [11,12]		Vitreal injections of soluble VEGFR1 inhibited ischemia-induced neo-vascularization in rat retina [13]. Note:VEGFR1 also binds PlGF	Human melanoma cells stably expressing *Vegf* antisense RNA formed small and poorly vascularized tumors in mice [14]
*Vegfr1*	*Vegfr1*^−/−^: lethal at E8.5 with excessive growth and disorganized vessels [15]. Reduced sprouting from embryonic stem cells [16,17]. VEGFR1 TK knockout: no effect on vascular development, but reduced macrophage migration [18]. Flt1 KD in zebrafish: increased intersegmental sprouting [19].	*Vegfr1* TK knockout: Impaired tumor metastasis by suppression of MMP-9 expression [20,21].		Blocking VEGFA and PlGF binding to VEGFR1: reduced tumor growth and vascularization [22,23]. Antisense mediated downregulation of VEGFR1 suppressed tumor growth in mice [24] Note that VEGFR1 is expressed by Leukocytes.
*Vegfr2*	*Vegfr2*^−/−^: lethal at E8.5–9.5; defective hematopoietic and endothelial cell differentiation [25].	NT	Ab-mediated VEGFR2 Neutralization led to retraction of tip cell filopodia and inhibited angiogenesis in the postnatal retina [9,26].	VEGFR2 block with small molecules or Abs suppressed endothelial growth and migration, induced apoptosis and regression of vessels in tumors [27,28,29]. Soluble VEGFR1 or neutralizing antibody: reduced tumor growth and metastasis [30,31].
*Vegfr3*	*Vegfr3*^−/−^: lethal at E10.5, defective arterial-venous remodeling of primary vascular plexus [32]. EC specific deletion of VEGFR3 results in excessive angiogenesis [33].	Excessive angiogenesis in tumor xenografts in endothelial specific VEGFR3 knockout mice [33].	VEGFR3 blocking Ab: reduced vascular density, number of branch points and sprouts in the postnatal retina [26].	VEGFR3 blocking Ab: reduced sprouting angiogenesis in tumor xenografts in mice [26,34].
*EphB4*	*EphB4*^−/−^ mice: lethal at E10.5 as a consequence of perturbed arterial-venous differentiation; arrest in cardiac development [35,36].	*EphB4^+/−^* mice: enhanced tumor growth in an intestinal tumor genesis model [37].	Blocking EphB4/EphrinB2 signaling by soluble EphB4 inhibited angiogenesis in murine Matrigel and corneal pocket assays [38].	Specific antibodies to EphB4, soluble EphB4 or small molecule inhibitors reduced tumor angiogenesis [38,39,40].
*EphrinB2*	*Efnb2*^−/−^: lethal at E11.5, defective maturation of arteries and veins into capillaries [36,41]. EphrinB2 PDZ signaling-deficient mice displayed reduced number of tip cells, fewer filopodia at the vascular front in retina. Mural cell specific inactivation of EphrinB2 resulted in defective microvessel architecture [42].	EphrinB2-PDZ signaling-deficient mice showed decreased tumor vascularization and reduced tumor growth [42].	NT	NT
*Neuropilin-1*	*Nrp1*^−/−^: lethal around E13, loss of filopodia extension in tip cells and impaired microvessel branching [43,44,45].	NT	Nrp-1 monoclonal Ab: reduced vascular sprouting and remodeling in the developing mouse retina [46].	Anti-Nrp-1 in combination with anti-VEGF Ab: reduced vessel density in tumor and exhibited additive effect on tumor suppression [46].
*Delta-like4*	Most Dll4^+/−^ mice die around E10.5 (strain dependent) with defective arteriogenesis and vascular remodeling [47,48,49]. Dll4^+/−^ or EC-specific ko display increased tip cell formation and branching [50,51,52,53]	Dll4 targeting increased tumor vascular sprouting and branching, but with poor perfusion and therefor suppressed tumor growth [54,55,56].	Antibody against Dll4 or soluble Dll4 promotes vascular sprouting and block artery development [56,57]. Dll4-Fc intraocular injections caused retinal hypersprouting [52]	Antibody against Dll4, soluble Dll4 or Dll4 vaccination increased non-functional tumor vessel growth and inhibited tumor growth [56,57,58].
*Delta-like1*	Dll1^−/−^:Lethal around E12 with sever hemorrhage [59]. Heterozygous Dll1 mice have impaired postnatal arteriogenesis [60].	NT	NT	NT
*Notch1*	*Notch1*^−/−^: lethal at E9.5 with disrupted vascular remodeling [61]. EC specific knockout of Notch1 increased tip cell formation [53].	Induced ablation of Notch1 in adult mice facilitated tumorigenesis in the skin [62]	Inhibition of Notch signaling by γ-secretase inhibitor increased tip cells and vessel branches [53].	γ-secretase inhibitor suppressed tumor growth by directly targeting tumor cells and inhibition of angiogenesis [63].
*Plexin D1*	*plexinD1*^−/−^:Perinatal lethality, abnormal cardiac development and defective intersomitic blood vessels and skeletal morphology [64,65].	NT	NT	NT
*Angiopoie-tins*	*Ang1*^−/−^: lethal at E12.5 with impaired vascular branching and remodeling [66].	NT	NT	Antisense *Ang1* RNA: Reduced xenograft tumor growth and angiogenesis [67]. Adenoviral mediated gene transfer of soluble Tie2 inhibited tumor growth and metastasis [68].
*Tie1*	*Tie1*^−/−^: lethal between E13.5 and birth with edema and hemorrhage; defective structural integrity of ECs [69]. Ang1 and Tie1 double knockout failed to establish the right hand side venous system [70].	NT	NT	NT
*Semaph-orins*	*Sema3a*^−/−^ (Nrp1 ligand) mice embryos showed disrupted vasculature [71]. *Sema3C*^−/−^: perinatal death, congenital cardiovascular defects with interruption of the aortic arch [72]. *Sema3E*^−/−^: disorganized embryonic intersomitic vessels [73].	Impaired maturation of tumor vessels in *Sema 4d*^−/−^ mice [74].	NT	Sema 3A administration reduced tumor vascularization [75]
*Unc5b*	*Unc5b*^−/−^: lethal from E12.5 (CD1 strain survives); enhanced filopodia formation and branching [76,77].	NT	Unc5b blocking Ab: Increased vascular density and sprouting of the postnatal retinal vasculature [77].	NT

Some genes in the table have not been discussed in the text. E, embryonic day; KD, knock down; NT, not tested; TK, tyrosine kinase; MMP, matrix metallo-proteinase; Ab, antibody; EC, endothelial cell; ko, knock out; KD, knock down.

### 1.2. Players in the Dynamic Establishment of the Vasculature

Blood vessels are built of ECs, supportive mural cells (pericytes and smooth muscle cells), and their shared basement membrane. In the developing embryo the main vessels are assembled by *in situ* differentiation of precursor cells in a process denoted as vasculogenesis [78]. From this primary vascular network new branches are formed through coordinated events of EC proliferation and migration, termed sprouting angiogenesis [79]. A multitude of signaling pathways are at play to balance the frequency of sprouting events and to guide the extending branch (reviewed in [79]). VEGF-A and -C and their receptors VEGFR1-3, the neuropilins (Nrps), the semaphorins (Sema), the eph and ephrins [42], the angiopoietins (Ang) and endothelial TEK tyrosine kinase receptors (Tie), Jagged1 and Delta like ligands (Dll)-1 and -4 and Notch-1 and -2 and VE-Cadherin are all required for correct temporal and spatial vascular patterning [80,81,82]. Many of these molecules are differentially expressed by subsets of ECs within the vascular sprout, demarking the cellular heterogeneity of the growing vasculature. Cells at the very front of sprouts with extensive actin rich protrusions and a certain gene expression profile are termed tip cells whereas the cells located just behind are referred to as stalk cells [9,83,84]. The tip cells are morphologically very similar to the axon-guiding growth cone and have in part similar functions, to lead the way [9,84,85,86]. Cells at the very tip of the sprout frequently express relatively higher levels of several proteins such as VEGFR-2, -3, Dll4, neuropilin-1, platelet-derived growth factor (PDGF)-B, Unc5b, EphrinB2 and Cxcr4a than their neighbors [83,87,88,89]. The differential proteome of each cell will set its potential in acquiring the tip cell position. In part these relations are manifested by EC to EC signaling via the Dll4-Notch1 pathway and its ability to regulate expression of the VEGFRs. However, none of these proteins are exclusively expressed by the leading cell. In accordance we recently demonstrated that the tip cell phenotype is transient and that cells can be over-taken by previous stalk cells [10]. This indicates that the “tip cell gene expression profile” may be switched on even before the cell has acquired the tip position and its characteristic morphology. It is commonly stated in publications that the tip cells are highly motile whereas the following stalk cells are not. However, recent data demonstrate that also cells within the stalk might be very motile [10,90]. Also, not all cells within the sprout migrate in a determined direction but seem to be chemokinetic, patrolling along the length of the forming sprout. The tip cells and the following one or two stalk cells lack pericyte coverage but secrete PDGFB that attracts mural cells by activation of their PDGFRβ [91,92]. EC-pericyte connections are required for vascular patterning and function as demonstrated by abnormal angiogenesis in pericyte-deficient mouse models [91]. As new branches are formed the basement membrane is deposited by endothelial tip- and stalk cells [93]. Therefore the growing front has an immature basement membrane that matures over time. The emerging sprout is transient and usually does not extend beyond the length of 150 μm *in vivo* before it connects to another vessel or anastomoses with an adjacent sprout to subsequently open up. Dysfunctional branches are then pruned and degraded as observed by live imaging or by the presence of empty collagen IV sleeves [88,94,95]. This final selection of branches is regulated by several factors such as shear stress, VEGF levels, and activity within the Notch pathway [94,96]. For example VEGF inhibition led to increased intussusceptive pruning in the chicken chorioallantoic membrane [96]. These observations clearly demark an enormous flexibility of the developing vascular tree. The extent and functional relevance of cellular shuffling in angiogenesis is still unknown.

### 1.3. Vascular Dynamics in the Tumor Microenvironment, What Is Actually Known?

Current knowledge on the modes of angiogenesis in tumor models in mouse is limited and for human cancers essentially absent. It is evident however that the vasculature of human tumors has an abnormal architecture, similar to what is observed in mouse models. Furthermore, the developing sprouting vasculature and the tumor vasculature have common traits such as poorly defined arterial/venous identity [97], incomplete pericyte coverage [98,99], non-perfused regions [9] and abnormal basement membrane with non EC-associated collagen IV indicative of vessel pruning [8,100]. In tumors, vessels may form via different processes: sprouting, intussusception, co-option, vascular looping and cancer stem-like cell differentiation [2,3,4,5,6]. It is difficult to discriminate between these various processes by analysis of fixed tumor material. Intravital microscopy has been used since the 1930s to image the tumor vasculature and its dynamic nature [101]. However it is not until recently that improved microscopy techniques have allowed for more detailed information on vascular dynamics. Vajkoczy *et al*. [102] injected C6 glioma cell suspensions and multicellular spheroids into striated muscle and the cerebral cortex of mice. By *in vivo* imaging they described an initial migration of injected tumor cells along host vessels (not considered co-option), but as the tumor started to establish itself, sprouting angiogenesis was initiated. Continued observation revealed several dysfunctional vessels that over time were pruned and regressed. Kienast *et al*. [103] seeded human lung carcinoma or melanoma cells into the carotid artery and imaged metastasis growth using multiphoton laser scanning microscopy in a cranial window over several days. By expression of red fluorescent protein from tumor cells and intravenous injections of FITC-dextran they dynamically visualized the synchronous expansion of the tumor and its vasculature at high resolution. Whereas the vasculature of the lung-derived tumors was highly angiogenic already two days after injection, the melanoma-derived tumor vasculature displayed preferentially capillary loop formation, tortuous vascular structures and vasodilatation. In conclusion these studies demonstrate constant remodeling of the vasculature of injected tumors in mice. Since the vasculature was visualized by fluorescence in the blood stream, which does not allow visualization of EC protrusions, little information is given on the behavior at the cellular level. Experimental data indicate that mechanisms of tip/stalk selection are at play in at least certain tumor models. Noguera-Troise *et al.* demonstrated extensive filopodia rich sprouting blood vessels of subcutaneous Lewis lung tumors and C6 rat glioma tumors. These sprouts expressed high levels of Dll4, similar to sprouting vessels in development [54]. Furthermore detailed studies of pancreatic islet tumors in the RIP-Tag mouse model (Rat insulin promoter driving the large T antigen of the simian virus 40 giving rise to insulinomas) revealed extensive EC sprouts suggesting involvement of sprouting angiogenesis [8]. Also, ECs of orthotopic gliomas in mice have been shown to extend branches and filopodia [42].

The similarities between the angiogenic vasculature in development and in tumors suggest that cellular programs may be common. By comparing information on knockout phenotypes in development with the ones of tumors we likely improve our understanding of how the tumor vasculature takes shape. Below we describe molecular pathways primarily involved in sprouting angiogenesis and the process of tip/stalk selection.

## 2. Angiogenesis in Development and Cancer—Lessons from Gene and Drug Interventions

From genetic studies and drug intervention studies it is evident that drivers of angiogenesis are shared in development, physiology and cancer. As described above several receptor-ligand systems have been carefully analyzed with respect to sprouting angiogenesis in development. Comparing genetic and drug induced loss- and gain- of function of the developing versus the tumor vasculature provides potential information on the process of tumor angiogenesis [104].

### 2.1. The VEGFs and VEGFRs

To date, activation of VEGFR2 by the hypoxia inducible ligand VEGFA is considered the initiating and main driving event of angiogenesis (reviewed in [79,105,106,107]). The importance of this signaling system is demonstrated by the haploinsufficiency of VEGFA and the embryonic lethality as a consequence of either total VEGFR2 deletion or mutation of its tyrosine residue 1173 [11,12,25,108,109]. Over recent years its ability of inducing sprouting angiogenesis and EC migration has been shown in numerous studies. Conversely, inhibition of VEGFR2 phosphorylation by the tyrosine kinase inhibitor SU5416 reduced sprouting and filopodia formation of the intersomitic vessels in the zebra fish embryo [110]. Also, intraocular injections of either soluble VEGFR1, to sequester VEGFA, or a VEGFR2-blocking antibody, lead to reduced tip cell filopodia and vascular branching in the postnatal retina [9]. Naturally it has therefore been the main therapeutic target in disease, and blockage display dramatic effects on the morphology of the vasculature [104]. Studies of pancreatic islet tumors in the RIP-Tag mouse model indicate reduced endothelial processes and empty collagen “sleeves”, demarking a reduced angiogenic profile upon VEGF blockage [8]. *In vivo* imaging by Kienast *et al*. (see above) revealed inhibition of angiogenesis in lung metastasis in the brain by treatment with the VEGF-trap, bevacizumab [103]. Furthermore, the vasculature became “normalized” with increased pericyte coverage, reduced density and more regular sized vessels [111,112].

As discussed below VEGFR2 activity is modulated by several co-receptors, such as neuropilin1 and Heparan sulphate proteoglycans but also by the mode of internalization, in turn regulated by eph receptors and VE-Cadherins [42,113,114,115]. In addition to VEGFR2, ECs express VEGFR1 that binds VEGFA with high affinity. The extracellular domain of VEGFR1 is required for vascular development and patterning whereas signaling via its tyrosine kinases is not. VEGFR1 has a negative impact on sprout formation and reduced VEGFR1 levels potentiate the tip cell phenotype [10,19]. VEGFR1 is also positively regulated by notch signaling and possibly functions to shape gradients by sequestering VEGFA [116].

VEGFR3 is expressed by the developing blood vasculature but is almost restricted to the lymphatics after embryonic day (E)13.5 [117]. The receptor is however re-expressed in the blood vasculature of many tumors [26,118,119]. Apart from its crucial role in development of the lymphatic vasculature it is required for development of the blood vasculature [32]. In several publications VEGFR3 has been shown to be enriched in the sprouting tip region of the postnatal retina and also, like VEGFR2, to be negatively regulated by Notch signaling [50,120]. Inhibition by VEGFR3 blocking antibodies reduced filopodia and sprout formation in the developing retina and in tumors [33]. In accordance VEGFR3 has been shown to promote sprouting of the embryonic intersomitic vessels of the zebrafish [120]. However, EC specific deletion of VEGFR3 in mice leads to ectopic sprouting, excessive filopodia formation and increased vascular density of the hindbrain and retina [26]. Similarly the vasculature of syngeneic subcutaneously implanted Lewis lung carcinomas displayed extensive filopodia and branching upon EC-specific VEGFR3 deletion. Also, in a mosaic vasculature of wild type and VEGFR3^+/−^ cells, VEGFR3^+/−^ cells preferentially became tip cells. Thus VEGFR3 may act both in a pro- and anti-angiogenic fashion likely depending on the presence of VEGFR2 and balance of the ligands VEGF-A and -C [33,121].

### 2.2. Delta-Like, Jagged and Notches

As mentioned above the membrane bound ligand Dll4 and signaling by its receptor Notch1 restricts sprouting angiogenesis and filopodia formation in the developing zebrafish and in the postnatal retina [50,51,53,56,110,120]. Blockage of notch cleavage and signaling by the gamma secretase inhibitor DAPT or by administration of soluble Dll4 protein induced vascular branching *in vitro* and *in vivo*. Dll4 is thought to mainly signal to Notch located on adjacent cells and thereby regulates selection of stalk and tip cells. Clonal analysis of chimeric developing vasculature demonstrated that cells with relatively higher Dll4 expression than their neighbors acquired the tip position at high frequency [10]. Accordingly, cells with reduced Notch signaling capacity relative to their neighbor frequently adopted the tip cell position [10,53,120]. Another Notch ligand, Jagged-1, is preferentially expressed by stalk cells and suppresses the tip cell phenotype by inhibition of Dll4-mediated notch signaling. EC specific jagged-1 LOF leads to decreased vascular density and sprouting [50].

Interference with Dll4 in several tumor models recapitulates what has been reported in development [56,57,58]. Blocking Dll4 signaling by administration of Dll4-Fc protein gave rise to increased vascular density but reduced tumor growth. This controversy was explained by the low degree of perfusion, indicative of a dysfunctional hypersprouting vascular bed.

### 2.3. The Eph and Ephrins

Several studies have reported the importance of the Eph receptor tyrosine kinase receptors and their ligands, the ephrins, as guidance cues for the developing vasculature [35,36,87]. Mice lacking the intracellular PDZ domain of EphrinB2 display reduced number of tip cells and filopodia extensions of the post natal retinal vasculature. In the same study the authors demonstrate reduced vascular density and filopodia numbers in an orthotopic glioma tumor model. Mechanistically, loss of the PDZ domain reduces VEGFR2 internalization thereby reducing its activation [42]. These data suggest that EphrinB2-dependent tumor angiogenesis at least in part is mediated by sprouting angiogenesis. In an additional study by Wang *et al*. endothelial specific deletion of EphrinB2 leads to reduced retinal sprouting angiogenesis [41]. Here, the authors link reduced sprouting to reduced internalization and reduced signaling via VEGFR3 instead. In addition morpholino-induced knockdown of efnb2a in zebrafish inhibited intersomitic sprouting and endothelial filopodia extensions. The eph-ephrin-system is not exclusively involved in sprouting angiogenesis but also during angiogenesis in the yolk sac, and in arterial to venous specification in development, suggesting important roles in other modes of angiogenesis than sprouting [122].

### 2.4. The Angiopoietins and Ties

The angiopoietin/Tie2 signaling system is required for vascular remodeling, maturation and stabilization. Mice lacking Ang1 displayed abnormal vasculature with reduced branching and poor mural cell coverage [66]. Overexpression of Ang1 in the skin of mice results in hypervascularization [123]. Unlike the hyperpermeable vessels induced by VEGF, vessels induced by Ang1 overexpression are leakage-resistant [124]. Coexpression of Ang1 and VEGF led to abundant vessel growth without leakage [124]. These findings suggest synergic functions of Ang1 and VEGF in the development of mature and stable vessels. The mechanism may relate to the functions of Ang1 in formation of EC junctions, or its involvement in EC adhesion and mural cell recruitment. Studies on cultured ECs have shown that Ang1 decreases the basal phosphorylation level of VE-Cadherin and PECAM-1 and inhibits permeability [125]. Ang1 can also serve as an adhesive substrate and promote endothelial adhesion and migration on extracellular matrix in an integrin-dependent manner [126]. Mural cell recruitment induced by Ang1 is partially mediated by stimulation of monocyte chemotactic protein-1 expression [127]. Ang1 also up-regulates Dll4 expression thereby contributing to vascular quiescence [128]. Ang2, another ligand of the Tie2 receptor, can act as a natural antagonist for Ang1/Tie2 signaling. Overexpression of Ang2 inhibits angiogenesis in the mouse embryo [129]. Targeting of Ang2 in mouse blocked the natural hyaloid vessel regression, revealing its indispensable role in postnatal vascular remodeling and regression [130]. Ang2 is up-regulated in the tumor vessels and promotes vascular destabilization and regression, which facilitate VEGF-induced angiogenesis in the later stage [131]. However, both Ang1 and Ang2 can alter their role in angiogenesis. Ang1 suppresses angiogenesis in the mouse heart while Ang2 collaborates with VEGFA to promote angiogenesis [132]. It is reported that Tie2 can translocate to either cell-cell or cell-matrix contacts upon Ang1 stimulation and thereby induce different downstream pathways [133,134]. This could explain the two faces of Tie2 signaling; inducing vascular quiescent or angiogenesis. Tie2 signaling is maintained in a precise balance in normal tissue, which makes it a challenging target for anti-angiogenic therapy. However, it has been reported that inhibition of Tie2 signaling by antisense Ang1 RNA or soluble Tie2 can inhibit tumor growth by reducing tumor angiogenesis [67,68]. Drugs targeting Ang1/2 or Tie2 are currently in clinical cancer trials [135]. Tie1 is the homologue of Tie2, but remains an orphan receptor so far. Deletion of Tie1 results in lethality between E13.5 to birth depending on genetic background; edema and hemorrhage are observed in the *Tie1*^−/−^ mice [69]. Double knockout of Ang1 and Tie1 generates a phenotype of right-hand side venous system loss [70]. Discovery of Tie1 ligands in the future will be helpful to elucidate its detailed function.

## 3. Conclusions

Almost all the proteins engaged in the regulation of developmental angiogenesis are likewise involved in tumor vascularization in mouse models (Table 1). Despite conserved EC expression and regulation of most of these molecules the resulting tumor vasculature is in part dysfunctional. This phenomenon should therefore be attributed differences in the non-vascular tissue of the developing organism and the tumor, respectively. In development tissue boundaries are well organized and patterning is strictly controlled. For example the intersomitic vessels are clearly guided by extracellular matrix proteins deposited in the intersegmental space and astrocytes and Müller glia form a scaffold on which the endothelial plexus can grow in the post natal retina of mice. In the tumor environment, tissue organization is often lost and production of growth factors and extracellular matrix components is abundant. Low oxygen levels, high enzymatic activity and altered pH create a highly reactive microenvironment prone to feed vascular malformations. As a consequence the vascular integrity is lost with detachment of pericytes and leakage of plasma proteins into the tumor stroma. Fibronectin deposition enhance migration of myofibroblasts and infiltrating leukocytes [136]. The altered flow dynamics of the irregular vasculature may in itself feedback to promote vascular remodeling. In development it was demonstrated that tip cell filopodia was instantly retracted upon initiation of flow in the sprouting central arteries of the zebrafish hindbrain, demonstrating the power of shear forces [88]. Furthermore, poor delivery of blood borne molecules (*i.e*., sphingosine-1 phosphate, Gaengel *et al*. unpublished) involved in mediating vascular quiescence could further add to the hyper active vascular phenotype. Within this review we have described that molecules known to regulate sprouting angiogenesis also have similar functions in the vasculature of tumors as judged from knockouts and drug targeting. Despite these observations very limited data on the actual dynamics of the tumor vasculature exist. It is not known what mode of vascular expansion *i.e*., sprouting, intussusception, vascular looping or vascular mimicry that is dominating in tumor growth. It is reasonable to believe that these various routes are closely linked and they may even occur simultaneously. Technical advances in the field of microscopy and imaging offer great potential in resolving many of these questions [137,138,139].
